# Supplementary material for: Role of the Cyclooxygenase Pathway in the Association of Obstructive Sleep Apnea and Cancer
Source: J Clin Med. 2020 Oct 10;9(10):3237. doi: 10.3390/jcm9103237 (PMC7601393; doi:10.3390/jcm9103237)
Supplement: Supplementary file 1 [file jcm-09-03237-s001.pdf]

Table S1. Regulatory role of the COX-2/Prostaglandin E2 pathway of the immunoediting mechanism in cancer development.

Target	Effects of COX-2 and PGE2	References
CDA4+ and CD8+T cells	-PGE2 inhibits production and proliferation<br>-PGE2 promotes differentiation in Treg	[84,85]
Th1	-PGE2 inhibits TH1 proliferation	[86]
Treg	-COX-2 expression regulates Treg induction<br>-COX-2 via Treg inhibits Th1 activation<br>-COX-2 inhibition attenuates Treg activation	[87–90]
NK	-PGE2 inhibits migration, cytotoxic effects and secretion of IFN-gamma.<br>-COX-2 inhibitors re-establish NK functions	[93–98]
DC	-PGE2 suppress tumor-infiltrating DC activation<br>-PGE2 promotes differentiation DC into monocytic MDSC and facilitates immune evasion<br>-PGE2 enhances IL-10 production and down-regulates DC functions<br>-PGE2 inhibits secretion of IFN-alpha which results in the induction of Th2 cytokines and reduction of Th1 cytokines	[99–104]
Macrophages	-COX-2 inhibition shifts the phenotype of TAM from M2 to M1	[106]
MDSCs	-COX-2 inhibition prevents local and systemic expansion<br>-PGE2 maintains the suppressive activity of cells	[108–113]
Angiogenesis	-Overexpression of COX-2 induces production of VEGF<br>-PGE2 stimulates VEGF expression	[115–119]
Wnt/Beta-Catenin pathway	-COX-inhibition attenuates signalling transmission	[144]
Exosomes	-COX-2 inhibition and antagonist of EP2 and EP4 receptors attenuates the inhibitory effects of exosomes over NK cytotoxic activity	[142]

References:

84. Harris, S.G.; Padilla, J.; Koumas, L.; Ray, D.; Phipps, R.P. Prostaglandins as modulators of immunity. Trends Immunol. 2002, 23, 144–150, doi:10.1016/s1471-4906(01)02154-8.

85. Mougiakakos, D.; Johansson, C.C.; Trocme, E.; Economou, M.A.; Larsson, O.; Seregard, S.; Kiessling, R.; All-Ericsson, C. Intratumoral forkhead box P3-positive regulatory T cells predict poor survival in cyclooxygenase-2-positive uveal melanoma. Cancer 2010, 116, doi:10.1002/cncr.24999.

86. Ikegami R, Sugimoto Y, Segi E, Katsuyama M, Karahashi H, Amano F, Maruyama T, Yamana H, Tsuchiya S, Ichikawa A. Treg Expression Of Prostaglandin E2 Receptors EP2 And EP4 And Their Different Regulation By Lipopolysaccharide In C3H/Hen Peritoneal Macrophages. J Immunol 2011,30,449-463.

87. Mahic, M.; Yaqub, S.; Johansson, C.C.; Taskén, K.; Aandahl, E.M. FOXP3+CD4+CD25+ Adaptive Regulatory T Cells Express Cyclooxygenase-2 and Suppress Effector T Cells by a Prostaglandin E2-Dependent Mechanism. J. Immunol. 2006, 177, 246–254, doi:10.4049/jimmunol.177.1.246.

88. Yuan, X.; Chen, L.; Li, M.-X.; Dong, P.; Xue, J.; Wang, J.; Zhang, T.-T.; Wang, X.-A.; Zhang, F.-M.; Ge, H.-L.; et al. Elevated expression of Foxp3 in tumor-infiltrating Treg cells suppresses T-cell proliferation and contributes to gastric cancer progression in a COX-2-dependent manner. Clin. Immunol. 2010, 134, 277–288, doi:10.1016/j.clim.2009.10.005.

89. Fontenot, J.D.; Gavin, M.A.; Rudensky, A.Y. Foxp3 programs the development and function of CD4+CD25+ regulatory T cells. Nat. Immunol. 2003, 4, 330–336, doi:10.1038/ni904.

90. Sahin, M.; Sahin, E.; Köksoy, S. Regulatory T cells in cancer: An overview and perspectives on Cyclooxygenase-2 and Foxp3 DNA methylation. Hum. Immunol. 2013, 74, 1061–1068, doi:10.1016/j.humimm.2013.05.009.

91. Yao, C.; Sakata, D.; Esaki, Y.; Li, Y.; Matsuoka, T.; Kuroiwa, K.; Sugimoto, Y.; Narumiya, S. Prostaglandin E2–EP4 signaling promotes immune inflammation through TH1 cell differentiation and TH17 cell expansion. Nat. Med. 2009, 15, 633–640, doi:10.1038/nm.1968.

92. Boniface K, Jensen K, Li Y, Blumenschein W, McGeachy M, McClanahan T, McKenzie RA, Kastlein RA, Cua DJ, de Waal Malefyt R. Prostaglandin E2 Regulates Th17 Differentiation And Function Through Cyclic AMP And EP2/EP4 Receptor Signalling. J. Exp. Med. 2009, 206, 535-548.
93. Bankhurst, A.D. The modulation of human natural killer cell activity by prostaglandins. J. Clin. Lab. Immunol. 1982, 7, 85–91.
94. Brunda, M.J.; Herberman, R.B.; Holden, H.T. Inhibition of murine natural killer cell activity by prostaglandins. J. Immunol. 1980, 124, 2682–7.
95. Holt, D.; Ma, X.; Kundu, N.; Fulton, A.M. Prostaglandin E2 (PGE2) suppresses natural killer cell function primarily through the PGE2 receptor EP4. Cancer Immunol. Immunother. 2011, 60, 1577–1586, doi:10.1007/s00262-011-1064-9.
96. Martinet L., Jean C., Dietrich D., Fournié J. J., Pouport R. PGE2 Inhibits Natural Killer And Gamma Delta T Cell Toxicity Triggered By NKR And TCR Through A Camp-Mediated PKA Type I-Dependent Signalling. Biochem Pharmacol 2010, 15, 838-845.
97. Kundu, N.; Ma, X.; Holt, D.; Goloubeva, O.; Ostrand-Rosenberg, S.; Fulton, A.M. Antagonism of the prostaglandin E receptor EP4 inhibits metastasis and enhances NK function. Breast Cancer Res. Treat. 2008, 117, 235–42, doi:10.1007/s10549-008-0180-5.
98. Kundu, N.; Walser, T.C.; Ma, X.; Fulton, A.M. Cyclooxygenase inhibitors modulate NK activities that control metastatic disease. Cancer Immunol. Immunother. 2005, 54, 981–987, doi:10.1007/s00262-005-0669-2.
99. Harizi, H.; Juzan, M.; Pitard, V.; Moreau, J.-F.; Gualde, N. Cyclooxygenase-2-Issued Prostaglandin E2Enhances the Production of Endogenous IL-10, Which Down-Regulates Dendritic Cell Functions. J. Immunol. 2002, 168, 2255–2263, doi:10.4049/jimmunol.168.5.2255.
100. Harizi, H.; Grosset, C.; Gualde, N. Prostaglandin E2modulates dendritic cell function via EP2and EP4receptor subtypes. J. Leukoc. Biol. 2003, 73, 756–763, doi:10.1189/jlb.1002483.
101. Yang L., Yamagata N., Yadav R, Brandon S., Courthey R.C., Morrow J.D., et al. Cancer-Associated Immunodeficiency And Dendritic Cell Abnormalities Mediated By The Prostaglandin E2 Receptor. J Clin Invest 2003, 111, 727-735.
102. Stock, A.; Booth, S.; Cerundolo, V. Prostaglandin E2 suppresses the differentiation of retinoic acid–producing dendritic cells in mice and humans. J. Exp. Med. 2011, 208, 761–773, doi:10.1084/jem.20101967.
103. Stock A, Booth S, Cerundolo V. Prostaglandin E2 Suppresses The Differentiation Of Retinoic Acid-Producing Dendritic Cells In Mice And Humans. J Exp Med 2011, 208, 761-73.
104. Fabricius D, Neubauer M, Mandel B, Schütz Z.C., Viardot A.,Volimer A.,et al. Prostaglandin E2 Inhibits IFN-Alpha Secretion And TH1 Costimulation By Human Plasmocytoid Dendritic Cells Via E-Prostanoid 2 And E-Prostanoid 4 Receptor Engagement. J Immunol 2010, 184, 677-684.
105. Eruslanov, E.; Daurkin, I.; Ortiz, J.; Vieweg, J.; Kusmartsev, S.; Ortiz, G. Pivotal Advance: Tumor-mediated induction of myeloid-derived suppressor cells and M2-polarized macrophages by altering intracellular PGE2 catabolism in myeloid cells. J. Leukoc. Biol. 2010, 88, 839–848, doi:10.1189/jlb.1209821.
106. Na, Y.-R.; Yoon, Y.-N.; Son, D.-I.; Seok, S.H. Cyclooxygenase-2 Inhibition Blocks M2 Macrophage Differentiation and Suppresses Metastasis in Murine Breast Cancer Model. PLOS ONE 2013, 8, e63451, doi:10.1371/journal.pone.0063451.
107. Eruslanov, E.; Kaliberov, S.; Daurkin, I.; Kaliberova, L.; Buchsbaum, D.J.; Vieweg, J.; Kusmartsev, S. Altered Expression of 15-Hydroxyprostaglandin Dehydrogenase in Tumor-Infiltrated CD11b Myeloid Cells: A Mechanism for Immune Evasion in Cancer. J. Immunol. 2009, 182, 7548–7557, doi:10.4049/jimmunol.0802358.
108. Sinha, P.; Clements, V.K.; Fulton, A.M.; Ostrand-Rosenberg, S. Prostaglandin E2 Promotes Tumor Progression by Inducing Myeloid-Derived Suppressor Cells. Cancer Res. 2007, 67, 4507–4513, doi:10.1158/0008-5472.can-06-4174.
109. Fujita, M.; Kohanbash, G.; Fellows-Mayle, W.; Hamilton, R.L.; Komohara, Y.; Decker, S.A.; Ohlfest, J.R.; Okada, H. COX-2 Blockade Suppresses Gliomagenesis by Inhibiting Myeloid-Derived Suppressor Cells. Cancer Res. 2011, 71, 2664–2674, doi:10.1158/0008-5472.can-10-3055.
110. Obermajer, N.; Muthuswamy, R.; Lesnock, J.; Edwards, R.P.; Kalinski, P. Positive feedback between PGE2 and COX2 redirects the differentiation of human dendritic cells toward stable myeloid-derived suppressor cells. Blood 2011, 118, 5498–5505, doi:10.1182/blood-2011-07-365825.

111. Mao Y, Pschke I, Wennerberg E, Pico de Coaña, Egyhazi Brage S, Shuchultz I, et al. Melanoma-Educated CD14+ Cells Adquire A Myeloid-Derived Suppressor Cell Phenotype Through COX-2-Dependent Mechansims. Cancer Res 2013, 73, 3877-3887.
112. Cheon, H.; Rho, Y.H.; Choi, S.J.; Lee, Y.H.; Song, G.G.; Sohn, J.; Won, N.H.; Ji, J.D. Prostaglandin E2 augments IL-10 signaling and function. J. Immunol. 2006, 177, 1092–1100, doi:10.4049/jimmunol.177.2.1092.
113. Vasquez-Dunddel, D.; Pan, F.; Zeng, Q.; Gorbounov, M.; Albesiano, E.; Fu, J.; Blosser, R.L.; Tam, A.J.; Bruno, T.; Zhang, H.; et al. STAT3 regulates arginase-I in myeloid-derived suppressor cells from cancer patients. J. Clin. Investig. 2013, 123, 1580–1589, doi:10.1172/jci60083.
114. Xue X, Shah YM. Hypoxia-Inducible Factor-2 A Is Essential In Activating The COX2/Mpges-1/PGE2 Signalling Axis In Colon Cancer. Carcinogenesis 2018, 11, 163-169.
115. Lee, J.J.; Natsuizaka, M.; Ohashi, S.; Wong, G.S.; Takaoka, M.; Michaylira, C.Z.; Budo, D.; Tobias, J.W.; Kanai, M.; Shirakawa, Y.; et al. Hypoxia activates the cyclooxygenase-2–prostaglandin E synthase axis. Carcinog. 2009, 31, 427–434, doi:10.1093/carcin/bgp326.
116. Greenhoug A., Smartt H.J.M., Moore A.E., Roberts H.R., Williams A.C., Paraskeva C., et al. The COX-2/PGE2 pathway: key roles in the hallmarks of cancer and adaptation to the tumour microenvironment. Carcinogenesis 2009, 30, 377-386.
117. Fukuda, R.; Kelly, B.; Semenza, G.L. Vascular endothelial growth factor gene expression in colon cancer cells exposed to prostaglandin E2 is mediated by hypoxia-inducible factor 1. Cancer Res. 2003, 63, 2330–4.
118. Tsujii, M.; Kawano, S.; Tsuji, S.; Sawaoko, H.; Hori, M.; Dubois, R.N. Cyclooxygenase Regulates Angiogenesis Induced by Colon Cancer Cells. Cell 1998, 94, 273, doi:10.1016/s0092-8674(02)09420-5.
119. Jones, M.K.; Wang, H.; Peskar, B.M.; Levin, E.; Itani, R.M.; Sarfeh, I.J.; Tarnawski, A.S. Inhibition of angiogenesis by nonsteroidal anti-inflammatory drugs: Insight into mechanisms and implications for cancer growth and ulcer healing. Nat. Med. 1999, 5, 1418–1423, doi:10.1038/70995.
142 Deng, Z.-B.; Zhuang, X.; Ju, S.; Xiang, X.; Mu, J.; Liu, Y.; Jiang, H.; Zhang, L.; Mobley, J.; McClain, C.; et al. Exosome-like nanoparticles from intestinal mucosal cells carry prostaglandin E2 and suppress activation of liver NKT cells. *J. Immunol.* **2013**, *190*, 3579–89, doi:10.4049/jimmunol.1203170.
144 Araki, Y.; Okamura, S.; Hussain, S.P.; Nagashima, M.; He, P.; Shiseki, M.; Miura, K.; Harris, C.C. Regulation of cyclooxygenase-2 expression by the Wnt and ras pathways. *Cancer Res.* 2003, *63*, 728–34.
